# Increasing Reaction Rates of Water-Soluble Porphyrins for ^64^Cu Radiopharmaceutical Labeling

**DOI:** 10.3390/molecules28052350

**Published:** 2023-03-03

**Authors:** Mateusz Pęgier, Krzysztof Kilian, Krystyna Pyrzynska

**Affiliations:** 1 Heavy Ion Laboratory, University of Warsaw, Pasteura 5A, 02-093 Warsaw, Poland; 2 Faculty of Chemistry, University of Warsaw, Pasteura 1, 02-093 Warsaw, Poland

**Keywords:** porphyrins, copper-64, positron emission tomography

## Abstract

Searching for new compounds and synthetic routes for medical applications is a great challenge for modern chemistry. Porphyrins, natural macrocycles able to tightly bind metal ions, can serve as complexing and delivering agents in nuclear medicine diagnostic imaging utilizing radioactive nuclides of copper with particular emphasis on ^64^Cu. This nuclide can, due to multiple decay modes, serve also as a therapeutic agent. As the complexation reaction of porphyrins suffers from relatively poor kinetics, the aim of this study was to optimize the reaction of copper ions with various water-soluble porphyrins in terms of time and chemical conditions, that would meet pharmaceutical requirements and to develop a method that can be applied for various water-soluble porphyrins. In the first method, reactions were conducted in a presence of a reducing agent (ascorbic acid). Optimal conditions, in which the reaction time was 1 min, comprised borate buffer at pH 9 with a 10-fold excess of ascorbic acid over Cu^2+^. The second approach involved a microwave-assisted synthesis at 140 °C for 1–2 min. The proposed method with ascorbic acid was applied for radiolabeling of porphyrin with ^64^Cu. The complex was then subjected to a purification procedure and the final product was identified using high-performance liquid chromatography with radiometric detection.

## 1. Introduction

Porphyrins are natural macrocycles that are able to tightly complex metal ions. The application of porphyrins and their complexes with metal ions in non-invasive diagnostics is gaining increasing importance which allows early detection of disturbances or pathological changes caused by diseases [1,2]. Porphyrins are potent fluorophores, biologically compatible, and are known to preferentially accumulate in tumor tissue [1]. These compounds occurring in nature include the well-known, red-colored heme in hemoglobin, which is responsible for the transport of blood as well as the chlorophylls in some bacteria and plants which are utilized for photosynthesis [3]. Inspired by their role in nature, porphyrins and metalloporphyrins are used not only as agents for tumor diagnostics but also as photosensitizers in photodynamic therapy of cancer [4], contrast agents in magnetic resonance imaging [5], photodynamic antimicrobial chemotherapy [6], or enzyme models in bioinorganic chemistry [7].

Single-photon emission computed tomography (SPECT) and positron emission tomography (PET) are both highly sensitive nuclear imaging techniques, where small amounts of the radioactive tracer as prepared radiopharmaceutical are used to produce images of a given body part. SPECT uses gamma emitters injected into the bloodstream and subsequently taken up by certain tissues [8]. In healthy people, the distribution of the dossed radiopharmaceutical follows the physiological pattern, while in the case of some dysfunction, regions with increased or decreased content can be identified. In PET the short-lived radionuclides with the emission of positron are utilized [9]. Positron, after losing kinetic energy, interacts with an electron in an annihilation reaction, resulting in the emission of two gamma photons at an angle of 180 degrees. The distribution of the radiotracer within different tissues, according to the carrier molecule, can be calculated based on the determination of the point of that annihilation.

Generally, the radiopharmaceutical used in SPECT or PET consists of four parts: radionuclide, bifunctional ligand, linker, and target molecule. The target molecules (small proteins, peptides, fragments of monoclonal antibody) are able to transport complex radionuclides to the diseased tissue containing the appropriate target receptor. The complexes used in nuclear medicine have to exhibit high thermodynamic stability as a strong interaction between the metal and the ligand is necessary to ensure the complete complexation of the radionuclide and to limit transmetalation reaction with competing species [10,11,12]. Moreover, a radioisotope–ligand complex should exhibit high kinetic inertness to prevent the dissociation of the complex and thus, the release of the radionuclide in the biological medium. The bifunctional ligands are covalently attached to the targeting molecule either directly or through various linkers [3]. The linker can be a simple hydrocarbon chain to increase lipophilicity, a peptide sequence to improve hydrophilicity, or a poly(ethyleneglycol) linker to slow extraction by hepatocytes. The selection of suitable chelators can facilitate the development of an effective PET imaging probe by improving targeting properties. Thus, there is a continuous need for new efficient chelators that can satisfy all the requirements.

The choice of radionuclide for diagnostic radiopharmaceuticals in PET and SPECT depends on its nuclear properties (type of radiation, half-life, energy) as well as radionuclide production, the conditions for radiolabeling, and specific activity. ^64^Cu, with its half-time of 78.4 h, low positron energy (653 keV), and a short average tissue penetration range (0.7 mm), has received attention for radiopharmaceutical development due to its favorable nuclear decay properties that make it useful in the labeling of antibodies for immuno-PET applications [13]. It undergoes multiple decay paths, allowing not only PET imaging through positron emission but also offering the possibility of treatment due to the emission of β‾ radiation (theranostics) [13,14]. DOTA (1,4,7,10-tetra- azacyclododecane-1,4,7,10-tetra-acetic acid) derivatives are mostly used to synthesize bifunctional ligands able to tightly bind the copper ions [15,16]. Porphyrins offer potential for application in nuclear medicine techniques, as ^64^Cu is a potent radionuclide that can serve as a diagnostic and therapeutic agent. With their natural affinity for tumor tissues, porphyrins can also act as targeting molecules for ^64^Cu, which can efficiently deliver it to pathological tissues. As potent complexing ligands able to tightly bind ^64^Cu ions, porphyrins can serve in radiopharmaceutical synthesis [2].

Porphyrins are known for forming very stable complexes with metal ions, which is advantageous in view of possible radiopharmaceutical applications [2,10]. Chelating properties are not significantly affected by the type and number of substituents in the porphyrin ring, which allows the choice of a ligand with appropriate characteristics, such as hydrophilicity/hydrophobicity or partition coefficient octanol/water, for the intended purpose of imaging. The main drawback of their coordination reaction is relatively poor kinetics at room temperature, thus heating under reflux conditions was required over an extended period of time [17]. The rate-limiting steps are determined by the kinetic barrier of the metal insertion step into the planar porphyrin structure and its relative rigidity.

To increase the rate of the formation of metalloporphyrins with medium-sized ions (e.g., Cu(II)) different approaches have been adopted. The rate-limiting step in the metalation of these ions is the deformation of the porphyrin ring. One of the interesting methods is the use of large ions (e.g., Hg(II), Pb(II), Cd(II)) that relatively easily form coordination complexes [18,19,20]. As these cations are too big to be incorporated completely into a porphyrin ring, they form a complex in which the ion “sits” on the top of the porphyrin macrocycle. It causes much faster distortion of the originally planar ring of the porphyrin ligand and allows relatively easy access for smaller ions (e.g., Cu(II)) from the other side. Yet, as far as the radiopharmaceutical application is concerned, the use of these highly toxic metal ions during synthesis is unfavorable. Another way to implement this approach is to use a reducing agent for Cu(II) such as ascorbic acid, glutathione, or hydroxylamine [18,21].

Using a microwave synthesis unit, higher radiochemical yields can be achieved in shorter reaction time and small quantities of solvents employed [22,23,24]. Mechanochemistry and ultrasounds have been also recently proposed for the development of a new method for metalloporphyrin synthesis [25,26,27]. The synthesis of metal complexes under mechanochemical action requires the use of excess metal salt and the addition of NaOH solution to achieve moderate yields. Under ultrasound irradiation using water as a solvent, only an alkaline medium is necessary. The solventless mechanochemical synthesis of copper(II) complex of 5,10,15,20-tetrakis(N-methylpyridinium-4-yl)porphine tetraiodide (Cu-TMPyP) was obtained in 45% yield after 60 min of milling [26]. The synthesized metalloporphyrin required purification to remove the excess metal salt through exclusion chromatography using water as an eluent, followed by water evaporation. The Cu-TMPyP complex under 30 min ultrasound was obtained in 79% yield (determined by HPLC) and 53% isolated yield.

The aim of this work was the optimization of the synthesis of copper complexes with water-soluble porphyrins with cationic and anionic functional groups for potential application in radiopharmaceutical synthesis. Efforts were focused on developing the fastest possible method that would meet the requirements of labeling with short-lived copper isotopes used in PET. An optimized method was applied for the synthesis of the ^64^Cu–porphyrin complex.

## 2. Results and Discussion

**Figure 1:**
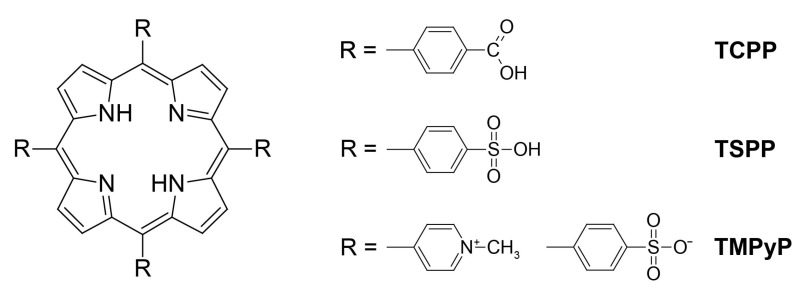
Structures of studied porphyrins.

Studied porphyrins included 5,10,15,20-tetrakis(N-methylpyridinium-4-yl)porphine tetratosylate (TMPyP), 5,10,15,20-tetrakis(4-sulfonato phenyl)porphyrin (TSPP) and for microwave synthesis also 5,10,15,20-tetrakis(4-carboxy phenyl)porphyrin (TCPP) (Figure 1).

**Figure 2:**
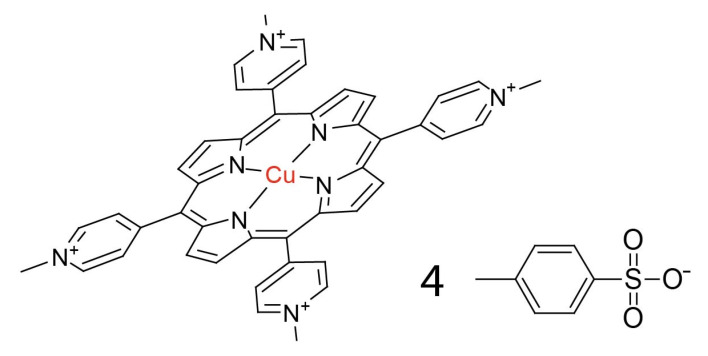
Structure of the copper(II) porphyrin complex on the example of Cu-TMPyP.

Electronic spectra of studied porphyrins are typical for this group of compounds [28,29]. The spectrum consists of the most intense band named the Soret band coming from the S_0_-S_2_ transition and four Q bands corresponding to split S_0_-S_1_ transitions. The formation of porphyrin complexes (structure presented in Figure 2) causes characteristic changes in electronic spectra. The number of Q bands decreases as symmetry break caused by core nitrogen protons no longer exists. The presence of metal ions also causes shifts of Soret and remaining Q bands. In the case of copper complexes, the shift in the Soret band is relatively small. Shifted Q(0,1) band is characteristic of the Cu complex of all studied porphyrins.

**Figure 3:**
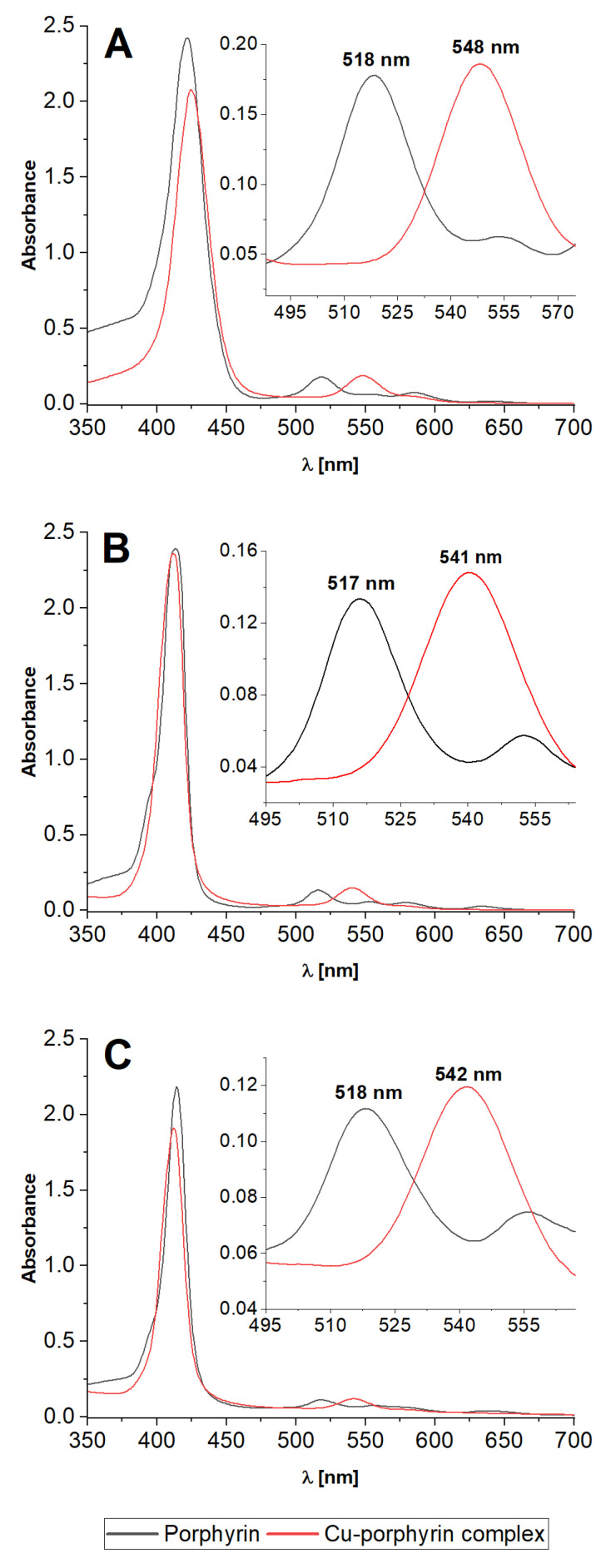
Spectra of studied porphyrins and corresponding Cu complexes: (**A**) TSPP, (**B**) TMPyP, (**C**) TCPP. [Cu-porphyrin] = [Porphyrin] = 10^−5^ mol L^−1^; pH 9.

Spectra presented in Figure 3 are typical for unprotonated porphyrins (pH 9). They are almost identical to those at pH 7 (with a minimal decrease in absorbance). The change in spectrum in the acidic region refers to the protonation of porphyrin with average pKa~5, depending on functional groups attached to the porphyrin ring. In the case of TMPyP, protonation is not yet visible in the spectrum at pH 4. However, both TSPP and TCPP become protonated at pH 4 as the Soret band is shifted to a higher wavelength and the intensity order of Q bands is inverted. The intensity of all bands is generally higher than in neutral and alkali media. These spectral changes can be observed as a change in color to deep green and it makes spectrophotometry a convenient method for monitoring of porphyrin reaction course. Spectra of highly protonated porphyrins are presented in Figure S1. TCPP tends to precipitate from the solution.

Cu–porphyrin complexes were synthesized in a water environment at three different pH values: acidic pH 4 (acetate buffer), neutral pH 7 (phosphate buffer), and basic pH 9 (borate buffer) to find optimal conditions. Only in the case of TCPP at pH 4 porphyrin tended to precipitate from the solution, especially after heating, so these conditions were not included in studies. Application of a water environment is highly desirable for possible radiopharmaceutical synthesis, as organic solvents generally must be thoroughly separated from the product, which unnecessarily lengthens and complicates the purification step. Different pH values were tested as it has been proven that acid–base equilibria can strongly influence the complexation of porphyrins. The [Cu]:[Porphyrin] ratio for all syntheses was 1:1.

As porphyrins tend to form aggregates in solutions, a study had to be performed to define the appropriate concentration of reagents for spectrophotometric monitoring of complexation reactions. It was completed by recording spectra of porphyrin solutions at different concentrations and analysis of the dependence of absorbance vs. concentration. The deviations from linearity may suggest the formation of aggregates that could influence the identity of the final product, which is important in radiopharmaceutical synthesis and quality control. The high absorbance (well above 1 absorbance unit) of the Soret band made it unavailable to use the most intense porphyrin band, as linearity was quickly violated due to high absorption. For this reason, aggregation was studied by plotting the absorbance of the most intense band in the Q region of the porphyrin spectrum, namely Q1 for all except from TSPP at pH 4, where the Q1 band is virtually invisible and the Q4 band exhibits the highest absorption. Examples of fitted data are presented in Figure S2. Results show that in the studied concentration range, porphyrins show no signs of aggregation, as R^2^ for all the plots reaches at least 0.999. The only exception is TSPP at pH 4, where the upper limit is 1.5 × 10^−5^ mol L^−1^. Thus, 10^−5^ mol L^−1^ has been chosen as the optimal concentration for all further studies, as it is far enough from the upper limit and made it possible to study even small changes in the spectrum.

**Figure 4:**
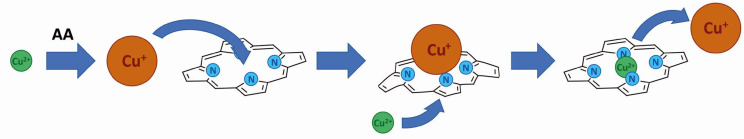
Overview scheme of synthesis of Cu(II)–poprhyrin complex involving formation of complex with large ions generated by reduction of Cu^2+^ ion to Cu^+^ ion with ascorbic acid (AA).

Acceleration of the synthesis of Cu(II)–porphyrin complex with the use of large ions can also be realized with the use of a reducing agent [19]. As far as the radiopharmaceutical application is concerned the choice of an acceptable reductant is important. In our study, ascorbic acid (AA) was employed (Figure 4). The reaction was conducted at a sufficiently high pH to maintain a deprotonated porphyrin ring. In the first rapid step, copper(II) is reduced to copper(I). As the ionic radius of Cu^+^ (96 pm) is larger than that of Cu^2+^ (72 pm), a complex with Cu^+^ ions remaining out of the ligand plane is formed. This leads to the deformation of the porphyrin ring. Subsequent incorporation of Cu^2+^ ion involves more favorable kinetics leading to almost immediate reaction in optimal conditions.

**Figure 5:**
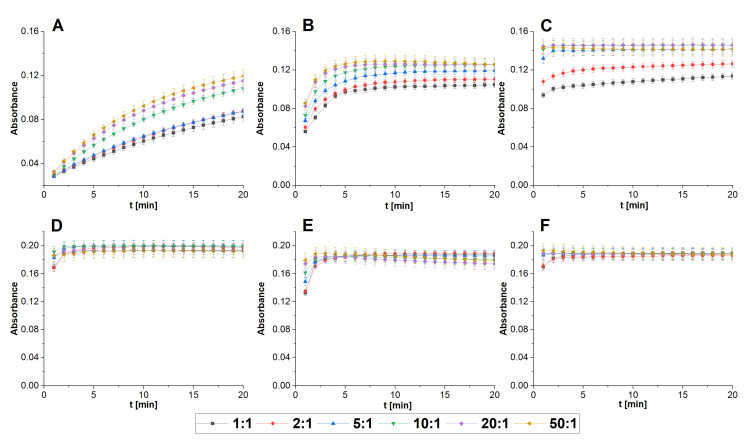
Complexation of Cu with porphyrins in a presence of AA at various [AA]:[Cu] ratios: (**A**) TSPP (pH 4); (**B**) TSPP (pH 7); (**C**) TSPP (pH 9); (**D**) TMPyP (pH 4); (**E**) TMPyP (pH 7); (**F**) TMPyP (pH 9). [Cu^2+^] = [TSPP] = [TMPyP] = 10^−5^ mol L^−1^.

Synthesis of Cu-TSPP complex in the presence of ascorbic acid (AA) as a reducing agent was running at the highest rate at pH 9 (Figure 5). Since the [AA]:[Cu] ratio equals to 5:1. maximum of complex absorbance is reached in 1 min. In the case of pH 7, a 20-fold excess of AA is needed to reach a maximum in 10 min or 50-fold (maximum is reached in 8 min). Reaction at pH 4 is the slowest and 20 min is not enough for complete synthesis of the Cu-TSPP complex. The reaction rate for TMPyP is generally higher than for TSPP with maximum absorbance reached at all pH values. Similarly to TSPP, at pH 9 the reaction is the fastest with an almost instant process, and maximum absorbance is reached between mixing reagents and recording first spectrum.

Contrary to TSPP, complexation at pH 4 is only slightly inferior to pH 9. From the 10-fold excess of AA upwards the reaction time is about 1 min. For both porphyrins, basic pH led to the deprotonation of pyrrolic nitrogen atoms, which besides the SAT mechanism additionally promoted a high reaction rate of complexation. Strong differences in the behavior of both porphyrins at pH 4 come from the stronger protonation of the porphyrin ring in the case of TSPP. TMPyP remains in a more reactive form with weakly bound protons that can easily be substituted by the incorporation of copper into the π system of porphyrin molecules. As for the other porphyrins, in the case of TCPP, which was the object of the previous study [19], the complexation at pH 9 is the fastest. An almost immediate reaction was achieved with a 10-fold excess of AA. For all studied porphyrins, a significant increase was observed, as the reaction time at room temperature without AA was more than 100 min (Figure S3).

**Figure 6:**
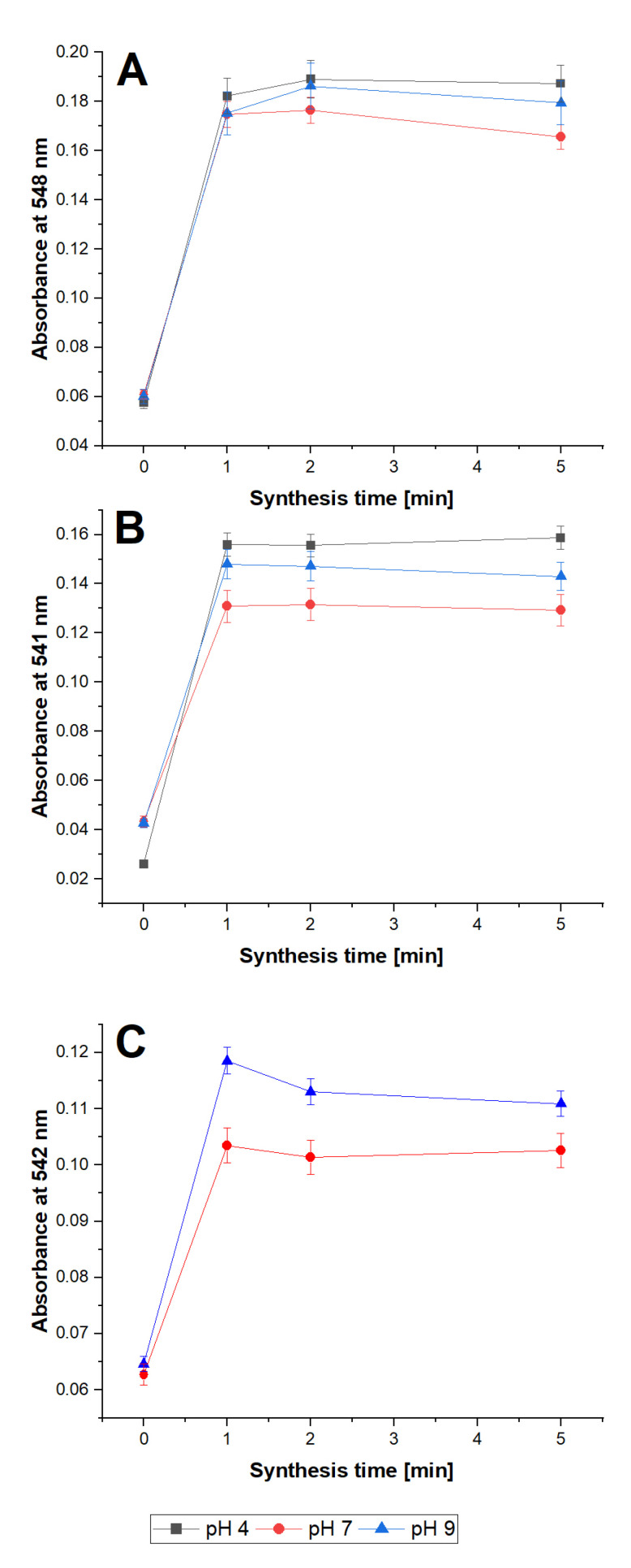
Microwave-assisted synthesis of Cu complex with: (**A**) TMPyP, (**B**) TSPP, (**C**) TCPP. [Cu^2+^] = [TSPP] = [TMPyP] = [TCPP] = 10^−5^ mol L^−1^.

The porphyrin ring is notably resistant to harsh conditions, such as high temperatures. For this reason, to increase the reaction rate microwave-assisted synthesis was applied. Microwave heating provides uniform energy transfer that prevents samples from overheating near reaction vessel walls and the possibility of precise control of reaction parameters. In order to maximize microwave energy, transfer continuous air cooling of the reaction vessel was applied. This allowed for a reduction of the reaction time to a minimum, which is vital for possible radiopharmaceutical synthesis. The obtained results are presented in Figure 6.

Microwave heating drastically reduced the time required for the synthesis of the Cu–porphyrin complex without the addition of any other chemicals, which is important in pharmaceutical preparations. Generally, 1 min was enough to reach the maximum absorbance of the corresponding complex. Only in the case of TMPyP after 2 min small increase was observed. Further elongation of synthesis did not cause any positive change. In the case of Cu-TCPP, a decrease in complex absorbance was noticeable, which shows the limited stability of the product. Compared to conventional heating, where in many cases 60 min was necessary to complete labeling [2], microwave-assisted synthesis showed a significant increase in reaction rate.

To assess the usefulness of the proposed complexation method, the reaction of TCPP with radioactive ^64^Cu (t_1/2_ = 12.4 h) was performed. As mentioned earlier, the complexation of macrocyclic ligands often requires harsh conditions that can be destructive for the targeting molecules due to irreversible modification of their structure. Thus, the rapid and stable complexation in mild conditions is a scientific challenge for the production of radiopharmaceuticals.

The ^64^Cu isotope was produced by proton irradiation of ^64^Ni in a medical cyclotron, then separated and finally dried in the form of ^64^CuCl_2_. Before further experiments, it was dissolved in deionized water. This form, without any purification, was used for labeling according to the procedure developed, achieving satisfactory yield with good radiochemical purity. Direct complexation resulted in a 78.7% yield at room temperature immediately. Further purification using solid phase extraction with an anion exchange column eliminated free ^64^Cu and increased the radiochemical purity up to 99.0%. It was confirmed by radio-HPLC where ^64^Cu was eluted in 2.3 min, while ^64^CuTCPP was eluted in 4.0 min referring to the radiometric detector signal (Figure S4).

**Figure 7:**
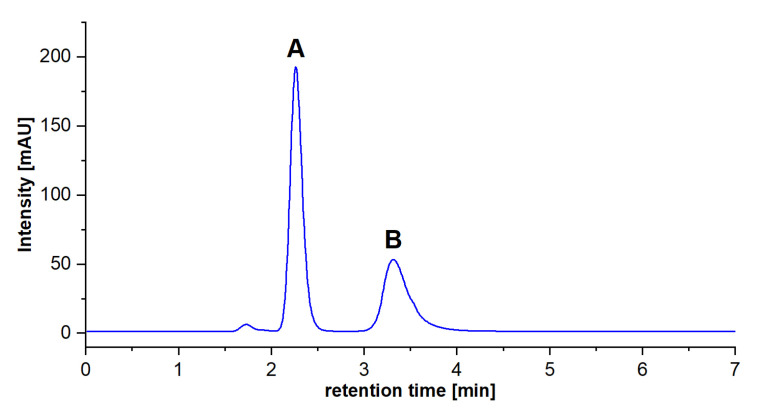
Separation of (**A**) TCPP (retention time 2.2 min) and (**B**) Cu-TCPP complex (retention time 3.3 min). Conditions: Phenomenex Gemini C18 column (150 mm × 4.0 mm i.d., 10 μm), mobile phase: 40:60 acetonitrile:0.05 mol L^−1^ CH_3_COOH/CH_3_COONa, pH 4.8, 1 mL min^−1^ flow rate.

Peaks are identified according to retention time (Figure 7) and specific spectra (Figure S5). This confirmed that the complexation of Cu(II) with the porphyrin ligand is fast and occurs within a single minute at pH = 9.

An interesting behavior was observed during purification on the anion exchange column. The complex could be eluted only in a specific sequence of eluents. After loading the column, water was used to remove water-soluble and cationic impurities (^64^Cu^2+^, buffers, and other metal ions). After that, gentle acidification (2 mL 0.1 mol L^−1^ HCl) of the column was required to remove the complex quantitatively (>98.5%) with 0.5 mL of ethanol. Any other sequences of eluents, including concentrated acids (1 mol L^−1^ HCl), acidification of the solution before the solid phase extraction step, and elution only with organic solvents, resulted in lower recovery or complex decomposition due to high concentration of acids. The explanation could be the mixed sorption mechanism; anion exchange for carboxylic groups combined with π–π interactions between the porphyrin ring and the solid phase of the column. Acidification breaks the ionic interactions and aprotic solvent releases the complex.

Another favorable phenomenon can be observed in the chromatographic separation of the post-reaction mixture. Other divalent metal ions may compete in the complexation process [30,31]. According to the supplier’s declaration for ^64^Cu^2+^, the solution may contain Ni(II) in a two-fold excess and Zn(II) ions in an approx. 40-fold excess vs. copper-64. As porphyrin ligands are used in excess they are not competitive. However, due to formal requirements in the development of radiopharmaceuticals, it may be necessary to remove these metal ions. The proposed HPLC method also allows for the separation of impurities in the form of Ni-TCPP and ZnTCPP complexes [28].

## 3. Reagents and Methods

### 3.1. Reagents

5,10,15,20-tetrakis(4-carboxyphenyl)porphyrin (TCPP) and 5,10,15,20-tetrakis(N-methylpyridinium-4-yl)porphine tetratosylate salt (TMPyP) were obtained from Sigma-Aldrich. 5,10,15,20-tetrakis(4-sulfonatophenyl)porphyrin tetraammonium salt was obtained from PorphyChem. Stock solutions (10^−3^ mol L^−1^) were prepared by dissolution of the appropriate amount of porphyrin in 5 mL of specific solvent: 3.59 mg of TCPP in 0.02 mol L^−1^ NaOH, 6.82 mg of TMPyP in 0.05 mol L^−1^ HCl and 5.02 mg of TSPP in deionized water.

Amounts of 0.1 mol L^−1^ borate buffer (pH 9) and phosphate buffer (pH 7) were prepared by dissolution of boric acid or sodium dihydrogen phosphate in water. Acetate buffer (pH 4) was prepared by dilution of 99.5% acetic acid with water. Further pH adjustment was performed using concentrated NaOH solution under control of Hanna HI 2210 pH meter (Hanna Instruments Inc., Smithfield, VA, USA) with combined glass electrode.

An amount of 1000 mg L^−1^ standard of Cu(II) ions as nitrates in 2–5% nitric acid (Merck) was further diluted with deionized water.

### 3.2. Study of Porphyrin Aggregation

Series of 2.5 × 10^−6^–2 × 10^−5^ mol L^−1^ porphyrin solutions at different pH were prepared and spectra were recorded. Then, linear fitting of the data using least squares method was performed and R^2^ factor was calculated to check the linearity of obtained results.

### 3.3. Direct Synthesis of Cu–porphyrin Complexes

Reactions were monitored with the Perkin Elmer Lambda 20 UV–VIS spectrophotometer (Perkin Elmer Inc., Waltham, MA, USA) with UV WinLab V2.85 software. Spectra were recorded in a range of 350–700 nm in polystyrene cuvettes of 1 cm length. To perform general synthesis at room temperature, 40 μL of stock porphyrin solution (10^−3^ mol L^−1^) was mixed with pH buffer and then 25 μL of stock Cu solution (100 mg L^−1^ in nitric acid) was added ([Cu] = [Porphyrin] = 10^−5^ mol L^−1^). Final volume of the sample was 4 mL. Then, spectra were recorded for 4 h.

### 3.4. Increasing Reaction Rate with the Use of Ascorbic Acid at Room Temperature

The study was conducted at three pH values—4, 7, and 9 at room temperature. Final concentrations of copper and porphyrins were 10^−5^ mol L^−1^. To study the influence of ascorbic acid (AA) on the complexation the concentration of AA was changed to achieve desired excess in relation to Cu. To carry out the study 40 μL of porphyrin was added to the proper buffer. Then, an appropriate volume of ascorbic acid and 25 μL of copper was added consecutively to reach desired excess of ascorbic acid in relation to Cu. Following excesses of AA were studied: 1, 2, 5, 10, 20, 50. For this reason, two stock solutions of ascorbic acid (AA) in acetate buffer were prepared by dissolution of solid ascorbic acid (Merck KGaA, Darmstadt, Germany): 0.05 mol L^−1^ for 50 and 20-fold excess of AA and 0.001 < for 1, 2, 5 and 10-fold excess of AA. Final concentrations of Cu(II) and porphyrin were 10^−5^ mol L^−1^. Reaction was monitored spectrophotometrically for 20 min. Due to low stability of AA, stock solutions wereprepared every day.

### 3.5. Microwave-Assisted Synthesis of Cu–porphyrin Complexes (without Ascorbic Acid)

Microwave-assisted synthesis was conducted without the addition of the AA due to its lack of thermal stability using CEM Discover SP microwave synthesizer (CEM Corporation, Matthews, NC, USA), which heated the sample with automatic real-time control of reaction parameters to prevent overheating and rapid increase in the pressure. Synthesis was performed in sealed 10 mL glass vessels with magnetic stirring. Temperature was set to 140 °C. The amounts of reagents were the same as for direct syntheses. In order to increase microwave power transfer continuous air cooling of the reaction vessel was applied. Reaction time was 1, 2, or 5 min. After cooling step sample was released and a spectrum was recorded.

### 3.6. Radiosynthesis of ^64^CuTCPP Complexes

^64^Cu was purchased from PET radioisotopes manufacturer (Voxel, Cracow, Poland) and was produced in ^64^Ni(p,n)^64^Cu reaction conducted on ALCEO solid target system (Comecer, Italy) coupled with GE PETrace 840 cyclotron (GE Healthcare Technologies Inc., Chicago, IL, USA). The requested activity was about 200 MBq in the form of dried ^64^CuCl_2_ reconstituted on-site with 1 mL of water. Stock porphyrin solution, buffers, and 0.05 mol L^−1^ ascorbic acid (Merck KGaA, Darmstadt, Germany) were prepared as described above. Water and phosphate-buffered saline (PBS) were of pharmaceutical grade. ^64^CuTCPP was synthesized at room temperature by mixing 200 μL of ^64^CuCl_2_ solution, 400 μL of TCPP, and 200 μL AA stock solutions, filled up with a borate buffer up to 5 mL, and vortexed. For final purification and formulation solution was loaded on anion exchange Waters Sep-Pak Accell Plus QMA column (Waters Corporation, Milford, CT, USA) with an ISM833 Ismatec peristaltic pump (VWR, Radnor, PA, USA) at 1 mL min^−1^, flushed with 5 mL of water at 2 mL min^−1^, acidified with 1 mL of 0.1 mol L^−1^ HCl and finally eluted with 0.5 mL of ethanol. After evaporation near dryness, small aliquot of PBS was added to reach expected activity concentration. An Atomlab 500 dose calibrator and wipe tester (Biodex, Shirley, NY, USA) was used for activity determination.

### 3.7. Determination of Radiochemical Purity of ^64^CuTCPP Complexes

Radiochemical purity was analyzed with the chromatography system Shimadzu AD20 with SPD-M20A UV–Vis photodiode array and radiometric (GabiStar, Raytest, Germany) detectors. The separation was performed on Phenomenex Gemini C18 column (150 mm × 4.0 mm i.d., 10 μm), with 40:60 acetonitrile: 0.05 M CH_3_COOH/CH_3_COONa adjusted to pH 4.8 as a mobile phase and 1 mL min^−1^ flow rate.

## 4. Conclusions

Studied porphyrins form stable complexes with copper(II) ions. The application of both methods, one with the use of ascorbic acid and the second with microwave heating drastically reduced the time required for the synthesis of Cu(II)–porphyrin complexes. In the case of the method with AA for all studied porphyrins, optimum conditions for fast and efficient complexation at room temperature comprise at least 10-fold excess of AA over Cu. Reactions with TSPP and TCPP should be conducted at pH 9, while for TMPyP pH 4 also offers a satisfying reaction rate. A method with microwave heating would be applicable only for thermally resistant molecules. If the porphyrin would act as a complexing agent attached to other, thermally sensitive targeting moiety, the method with the use of AA would be more convenient. Both proposed methods are convenient for application in radiopharmaceutical synthesis as they are one step, fast, and no toxic or harsh chemicals are used that would have to be separated. Additionally, they could be potentially adopted for the synthesis of radiopharmaceuticals labeled with short-lived copper isotopes (^60^Cu (t_1/2_ = 23.7 min), ^61^Cu (t_1/2_ = 3.3 h), and even ^62^Cu(t_1/2_ = 9.67 min)).

## Data Availability

Data generated during the study are available on request.

## Supplementary Materials

The following supporting information can be downloaded at: https://www.mdpi.com/article/10.3390/molecules28052350/s1, Figure S1. Spectra of protonated porphyrins. [TMPyP] = [TSPP] = [TCPP] = 10^−5^ mol L^−1^; pH 1.5.; Figure S2: Study of aggregation of porphyrins at different pH and concentrations. Wavelength of peak maximum is given in parentheses.; Figure S3. Complexation of Cu with porphyrins with and without the addition of AA (pH and [AA]:[Cu] ratio in brackets): (A) TSPP (pH 4, 50:1); (B) TSPP (pH 7, 50:1); (C) TSPP (pH 9, 10:1); (D) TMPyP (pH 4, 10:1); (E) TMPyP (pH 7, 20:1); (F) TMPyP (pH 9, 10:1). [Cu2+] = [TSPP] = [TMPyP] = 10^−5^ mol L^−1^.; Figure S4. Radio-chromatogram of raw product (A) and purified final product (B). Separation: Phenomenex Gemini C18 column (150 mm × 4.0 mm i.d., 10 μm), mobile phase: 40:60 acetonitrile:0.05 mol L^−1^ CH_3_COOH/CH_3_COONa pH 4.8, 1 mL min^−1^ flow rate.; Figure S5. Spectra of (A) TCPP and (B) Cu-TCPP recorded at retention times: 2.2 and 3.3 min, respectively. Separation: Phenomenex Gemini C18 column (150 mm × 4.0 mm i.d., 10 μm), mobile phase: 40:60 acetonitrile: 0.05 mol L^−1^ CH_3_COOH/CH_3_COONa pH 4.8, 1 mL min^−1^ flow rate.

## Sample Availability

Samples of the compounds TCPP, TSPP, TMPyP, and CuTCPP are available from the authors.
