# Supplementary material for: Increasing Reaction Rates of Water-Soluble Porphyrins for 64Cu Radiopharmaceutical Labeling
Source: Molecules. 2023 Mar 3;28(5):2350. doi: 10.3390/molecules28052350 (PMC10005645; doi:10.3390/molecules28052350)
Supplement: Supplementary file 1 [file molecules-28-02350-s001.zip › molecules-2175913-supplementary.pdf]

Supplementary material

Increasing of reaction rates of water soluble porphyrins for ^{64}Cu radiopharmaceutical labelling

Mateusz Pęgier [1,*], Krzysztof Kilian [1] and Krystyna Pyrzynska [2]

[1] Heavy Ion Laboratory, University of Warsaw, Pasteura 5A, 02-093 Warsaw, Poland
[2] Faculty of Chemistry, University of Warsaw, Pasteura 1, 02-093 Warsaw, Poland
* Correspondence: pegier@slcj.uw.edu.pl

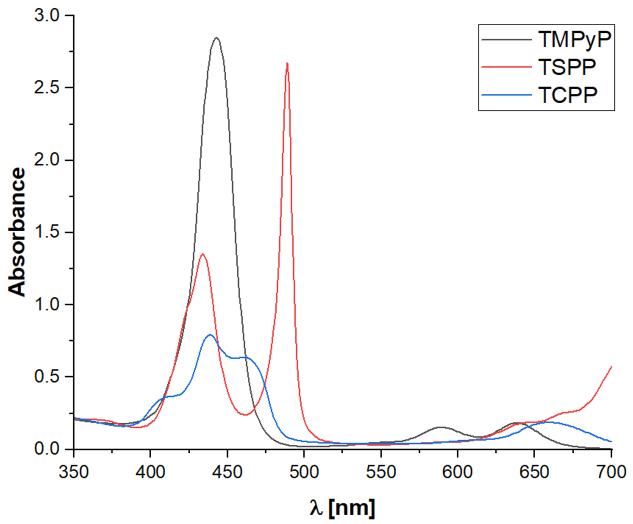

Figure S1. Spectra of protonated porphyrins. [TMPyP] = [TSPP] = [TCPP] = 10^{-5} mol L^{-1}; pH 1.5.

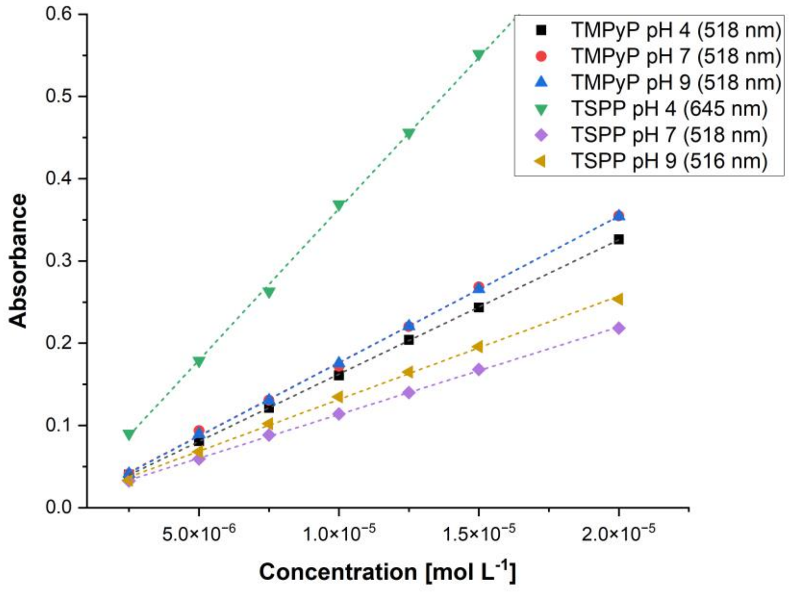

Figure S2. Study of aggregation of porphyrins at different pH and concentrations. Wavelength of peak maximum is given in parentheses.

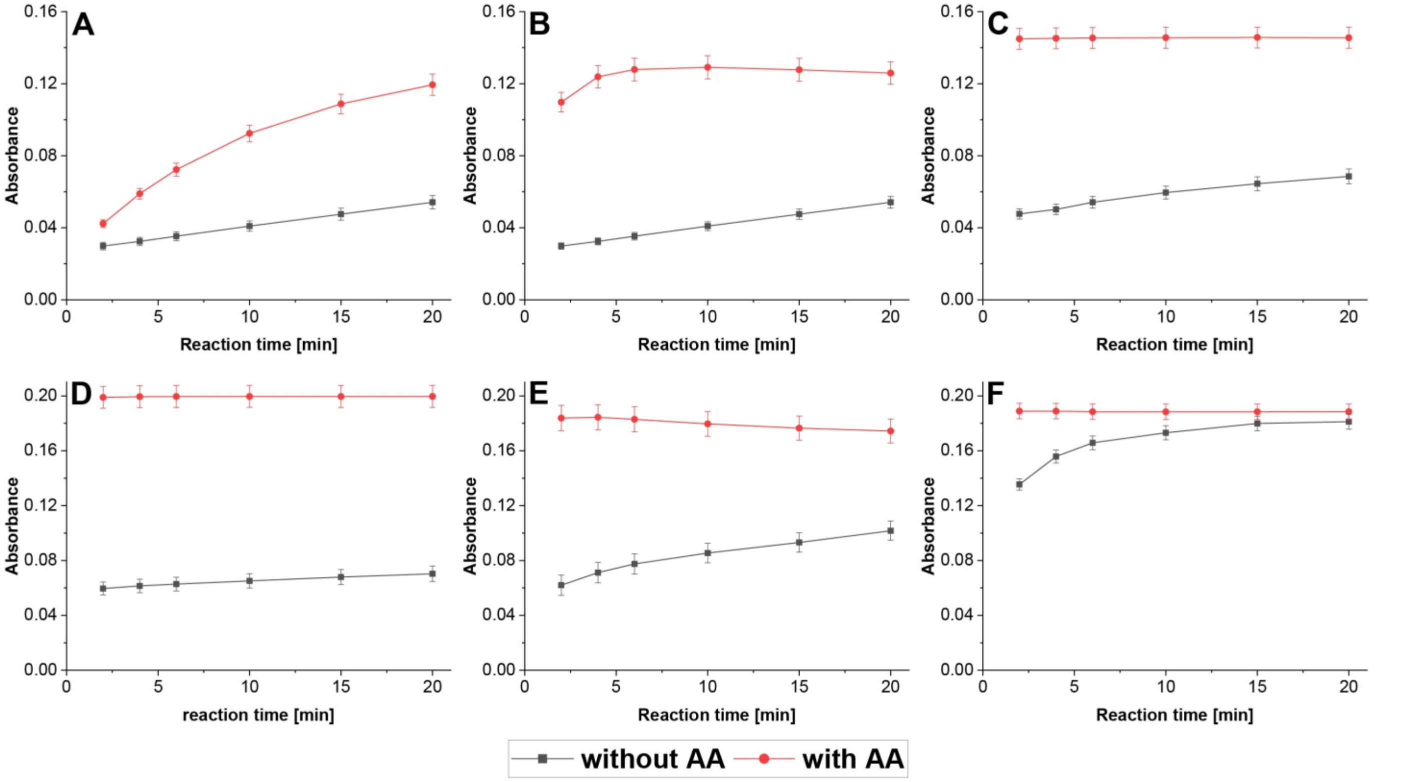

Figure S3. Complexation of Cu with porphyrins with and without the addition of AA (pH and [AA]:[Cu] ratio in brackets): (**A**) TSPP (pH 4, 50:1); (**B**) TSPP (pH 7, 50:1); (**C**) TSPP (pH 9, 10:1); (**D**) TMPyP (pH 4, 10:1); E – TMPyP (pH 7, 20:1); F – TMPyP (pH 9, 10:1). $[Cu^{2+}]$ = [TSPP] = [TMPyP] = 10^{-5} mol L^{-1}.

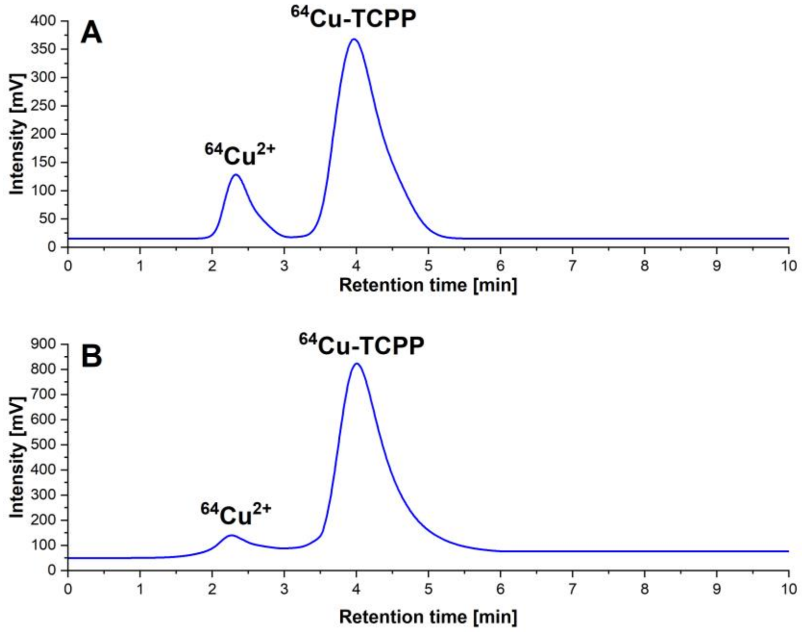

Figure S4. Radio-chromatogram of raw product (**A**) and purified final product (**B**). Separation: Phenomenex Gemini C18 column (150 mm × 4.0 mm i.d., 10 μm), mobile phase: 40:60 acetonitrile:0.05 mol L^{-1} CH_3COOH/CH_3COONa pH 4.8, 1 mL min^{-1} flow rate.

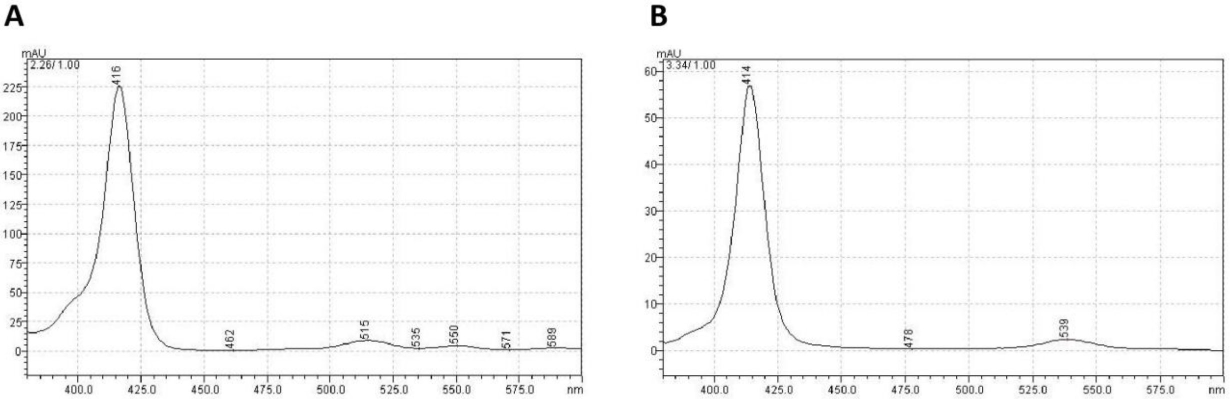

Figure S5. Spectra of (**A**) TCPP and (**B**) Cu-TCPP recorded at retention times: 2.2 and 3.3 min respectively. Separation: Phenomenex Gemini C18 column (150 mm × 4.0 mm i.d., 10 µm), mobile phase: 40:60 acetonitrile: 0.05 mol L^{-1} CH_3COOH/CH_3COONa pH 4.8, 1 mL min^{-1} flow rate.
